# A comparison of the prognostic value of composite ratios and cumulative scores in patients with operable rectal cancer

**DOI:** 10.1038/s41598-020-73909-0

**Published:** 2020-10-21

**Authors:** Ross D. Dolan, Muhammed Alwahid, Stephen T. McSorley, James H. Park, Richard P. Stevenson, Campbell S. Roxburgh, Paul G. Horgan, Donald C. McMillan

**Affiliations:** Academic Unit of Surgery, School of Medicine, University of Glasgow, Glasgow Royal Infirmary, New Lister Building, Glasgow, G4 0SF UK

**Keywords:** Cancer metabolism, Surgical oncology

## Abstract

The aim of this study was to directly compare the prognostic value of cumulative scores and composite ratios in patients with operable rectal cancer. Within a single surgical unit preoperative differential blood cell results including neutrophil (N), lymphocyte (L), monocyte (M) and platelet (P) counts, as well as CRP (C) and albumin (A) levels were recorded. These results were used to construct a series of composite ratios (NLR, PLR, LMR, CAR) and cumulative scores (NLS, PLS, LMS, NPS, mGPS). The relationship between composite ratios and the cumulative scores and clinicopathological characteristics, cancer specific survival (CSS) and overall survival (OS) were examined. A total of 413 patients were included. When adjusted for TNM stage, surgical approach, time of surgery and margin involvement mGPS (p < 0.05) was associated with CSS. In addition, most composite ratios/scores showed correlations with neoadjuvant therapy (p < 0.001). When a direct comparison between NPS (myeloid) and mGPS (liver) was carried out they showed similar associations with both CSS and OS. Therefore, both composite ratios and cumulative scores have been shown to be prognostic in patients with operable rectal cancer.

## Introduction

Annually there are 1.36 million new cases of colorectal cancer (CRC) worldwide^1^. In the UK specifically, colorectal cancer is the fourth most prevalent cancer and the second most common cause of cancer death^2^. Rectal cancers specifically account for 32% of male CRCs and 23% of female CRCs, making rectal cancer the most common GI cancer location below the ileo-caecal valve^2^. Overall death rates from colorectal cancer have fallen over the last decade, however it is still the case that approximately 40% of patients diagnosed colorectal cancer will die of their disease^1^.

Recently there has been an increase in the use of neoadjuvant chemoradiotherapy (CRT), particularly in patients with locally advanced or margin threatening rectal cancer^3^. This multi-modal approach, recommended by 2019 NICE guidelines has led to more patients being treated non-operatively with close follow up after complete pathological responses (cPR) to CRT^3^. Surgical resection still remains the primary curative treatment in the majority of cases however the treatment pathway has become more diverse. As a result, there is ongoing interest in the identification of patients at a higher risk of developing advanced disease who may benefit from surgical resection despite excellent responses to neoadjuvant therapy.

An increase in the systemic inflammatory response can identify patients at an increased risk of disease progression^4,5^. Indeed in two recent systematic reviews and meta-analyses Dolan and co-workers have shown that assessment of the systemic inflammation has prognostic value in gastrointestinal cancers^4,5^. Indeed, in both these reviews composite ratios and cumulative scores were the most widely validated means of assessing the systemic inflammatory response^4,5^. These scores and ratios were constructed from components of the differential white blood cell counts assessing the systemic inflammatory response in lymphoid tissue, and acute phase proteins assessing the inflammatory response in the liver^6^.

In a recent study in 801 patients undergoing surgery for colon cancer Dolan and co-workers directly compared cumulative ratios and composite scores including neutrophil/lymphocyte ratio (NLR) and neutrophil lymphocyte score (NLS), platelet/lymphocyte ratio (PLR) and platelet lymphocyte score (PLS), lymphocyte/monocyte ratio (LMR) and lymphocyte monocyte score (LMS) and C-reactive protein/albumin ratio (CAR) and modified Glasgow Prognositic Score (mGPS)^6^. This study showed that independent of TNM stage both composite ratios and scores had prognostic value^6^.

With the recent divergence in the management of rectal cancers with cPR away from surgical resection to a more conservative path with watchful waiting treatment regimens, it is increasingly important to accurately identify patients at higher risk of developing disseminated disease. To our knowledge the effectiveness of stratification of both composite ratios and cumulative scores has not been assessed in patients with rectal cancer. Therefore, the aim of the present study was to compare and contrast the prognostic value of composite ratios and cumulative scores, in patients undergoing surgery for rectal cancer.

## Patients and methods

Patients were identified from a prospectively maintained database of rectal cancer resections at Glasgow Royal Infirmary. Patients who met the following criteria were included. Firstly, those who up to 30 days prior to surgery had samples taken for serum CRP, albumin and differential blood cell counts; secondly, those who based on preoperative imaging and surgical findings were considered to have undergone potentially curative resection for rectal cancer between January 1997 and June 2015. Patients who did not have pre-operative CRP or albumin readings, whose cancers were related to inflammatory bowel disease and who underwent surgery for a limited resection or for a palliative intent were excluded^7^. Pre-operative colonoscopy, MRI rectum and CT thorax, abdomen and pelvis was performed in all patients. MRI liver and PET-CT was performed in select patients to confirm the extent of disease or if dubiety existed from standard imaging.

The majority of patients with moderate (cT3b or greater, suspicious lymph node within 1 mm of margin or extra-mural venous invasion) or high risk (tumour within 1 mm or margin, low tumours encroaching intersphincteric plane or with levator muscle involvement) of local recurrence were offered long-course neo-adjuvant chemoradiotherapy (50 Gy radiotherapy in 28 doses with 5-fluorouracil (5-FU) adjuvant chemotherapy) followed by TME surgery performed 8–12 weeks post completion.

Staging was based on the fifth edition of the TNM classification, with additional information being taken from the resectional pathological reports as required^8^. Following diagnosis, all patients were discussed at a multidisciplinary meeting. A decision on neo-adjuvant therapy or proceeding direct to surgery was made with the oncologists coordinating the administration of CRT. Post surgery, 5-FU adjuvant chemotherapy was offered to patients with stage III or high-risk stage II disease and no significant comorbidities.

Blood tests were collected and recorded prospectively as mentioned above. Composite ratios and scores were constructed as outlined in Table 1. Patients were routinely followed up for 5 years after surgery with 6 monthly CEA measurement, annual CT and colonoscopy at 5 years. Both the cancer registration system and Registrar General (Scotland) were crosschecked to ensure the cause of death was accurate. The censor date for this study was June 30th 2018. Cancer-specific survival (CSS) was measured from date of surgery until date of death from recurrent or metastatic rectal cancer. Overall survival (OS) was measured until the date of death from any cause. The West of Scotland Research Ethics Committee approved the study and all methods were performed in accordance with relevant guidelines and informed consent was obtained.

**Table 1:** Systemic inflammation based prognostic ratios and scores.

Neutrophil lymphocyte ratio(NLR)	
Neutrophil count:lymphocyte count	≤ 3
Neutrophil count:lymphocyte count	3–5
Neutrophil count:lymphocyte count	> 5
Neutrophil Lymphocyte Score (NLS)	
Neutrophil count ≤ 7.5 × 10^9^/l and lymphocyte count ≥ 1.5 × 10^9^/l	0
Neutrophil count > 7.5 × 10^9^/l and lymphocyte count ≥ 1.5 × 10^9^/l	1
Neutrophil count ≤ 7.5 × 10^9^/l and lymphocyte count < 1.5 × 10^9^/l	1
Neutrophil count > 7.5 × 10^9^/l and lymphocyte count < 1.5 × 10^9^/l	2
Platelet lymphocyte ratio (PLR):	
Platelet count:lymphocyte count	≤ 150
Platelet count:lymphocyte count	> 150
Platelet Lymphocyte Score (PLS)	
Platelet count ≤ 400 × 10^9^/l and lymphocyte count ≥ 1.5 × 10^9^/l	0
Platelet count > 400 × 10^9^/l and lymphocyte count ≥ 1.5 × 10^9^/l	1
Platelet count ≤ 400 × 10^9^/l and lymphocyte count < 1.5 × 10^9^/l	1
Platelet count > 400 × 10^9^/l and lymphocyte count < 1.5 × 10^9^/l	2
Lymphocyte monocyte ratio (LMR)	
lymphocyte count:monocyte count	≥ 2.40
lymphocyte count:monocyte count	< 2.40
Lymphocyte Monocyte Score (LMS)	
Lymphocyte count ≥ 1.5 × 10^9^/l and monocyte count ≤ 0.80 × 10^9^/l	0
Lymphocyte count < 1.5 × 10^9^/l and monocyte count ≤ 0.80 × 10^9^/l	1
Lymphocyte count > 1.5 × 10^9^/l and monocyte count > 0.80 × 10^9^/l	1
Lymphocyte count < 1.5 × 10^9^/l and monocyte count > 0.80 × 10^9^/l	2
Neutrophil Platelet Score (NPS)	
Neutrophil count ≤ 7.5 × 10^9^/l and platelet count ≤ 400 × 10^9^/l	0
Neutrophil count > 7.5 × 10^9^/l and platelet count ≤ 400 × 10^9^/l	1
Neutrophil count ≤ 7.5 × 10^9^/l and platelet count > 400 × 10^9^/l	1
Neutrophil count > 7.5 × 10^9^/l and platelet count > 400 × 10^9^/l	2
C-reactive protein albumin ratio (CAR)	
C-reactive protein:albumin	≤ 0.22
C-reactive protein:albumin	> 0.22
Modified Glasgow Prognostic Score (mGPS)	
C-reactive protein ≤ 10 mg/l and albumin ≥ 35 g/l	0
C-reactive protein > 10 mg/l and albumin ≥ 35 g/l l	1
C-reactive protein > 10 mg/l and albumin < 35 g/l l	2

### Statistics

CRP, albumin and differential blood cell count components were expressed as medians and ranges. Receiver operating characteristic (ROC) curve analysis was used to establish cut off values for individual ratios. The threshold values of such characteristics were based on the most prominent point on the ROC curve for ‘‘sensitivity’’ and ‘‘1-specificity,’’ respectively. The optimal threshold values were defined using the Youden index (maximum (sensitivity + specificity − 1)) and these were compared with published validated values to determine the value used in the subsequent analysis^9,10^. The area under the ROC (AUROC) curve was calculated. The relationship between NLR, PLR, LMR, CAR, NLS, PLS, LMS and mGPS and both cancer specific and overall survival was assessed using Cox proportional hazard regression to calculate hazard ratios (HRs) and 95% confidence intervals (95% CIs). The relationship between NLR, PLR, LMR, CAR, NLS, PLS, LMS and mGPS and patient clinicopathological characteristics was assessed using Pearson Chi-Square tests. In order not to adjust individual variables for multiple comparisons, the correlation of composite ratios and cumulative scores and clinicopathological characteristics, a p < 0.001 was considered significant across all variables. This p-value was similar to that when a Bonferroni correction was applied (0.05/23 = 0.00217). All analyses were performed using SPSS version 22.0 (IBM Corp, Armonk, NY).

## Results

A total of 413 patients met the inclusion criteria and were included in the final analysis (Table 2). The majority of patients were > 65 (69%), male (57%), BMI > 25 (54%), ASA ≥ 2 (87%), presented electively (99%), underwent open resection (83%), had surgery post 2005 (69%) and did not require adjuvant therapy (72%). The majority of patients had either TNM stage II or III disease (79%), moderate/well differentiated adenocarcinomas (91%, 18 of which had mucin lakes), venous invasion (57%), an R0 resection (89%), no peritoneal involvement (91%) and no tumour perforation (99%). Over the course of this studies follow up there were 115 (28%) cancer deaths and 197 (48%) deaths overall.

**Table 2:** The clinicopathological characteristics of patients undergoing surgery for rectal cancer (n = 413).

	Variables	n = 413 (%)
Age (years)	< 65	171 (41.4)
	65–74	143 (34.6)
	> 75	99 (24.0)
Sex	Female	176 (42.6)
	Male	237 (57.4)
BMI^a^	Underweight	33 (9.8)
	Normal	120 (35.7)
	Overweight	111 (33.0)
	Obese	72 (21.4)
Time of surgery	1997–2004	129 (31.2)
	2005–2015	284 (68.8)
ASA grade^b^	1	69 (23.2)
	2	128 (43.0)
	3	92 (30.9)
	4	9 (3.0)
Presentation	Elective	407 (98.5)
	Emergency	6 (1.5)
Type of surgery	Open	343 (83.1)
	Laparoscopic	70 (16.9)
Neoadjuvant therapy^c^	No	305 (75.3)
	Yes	100 (24.7)
Adjuvant therapy^d^	No	281 (71.5)
	Yes	112 (28.5)
T stage	1	42 (10.2)
	2	69 (16.7)
	3	251 (60.8)
	4	51 (12.3)
N stage	0	253 (61.3)
	1	114 (27.6)
	2	46 (11.1)
TNM stage	1	88 (21.3)
	2	161 (39.0)
	3	164 (39.7)
Differentiation^e^	Mod/well	375 (90.8)
	Poor	29 (7.2)
Venous invasion^f^	No	178 (43.4)
	Yes	232 (56.6)
Margin involvement^g^	No	364 (89.0)
	Yes	45 (11.0)
Peritoneal involvement^h^	No	371 (90.9)
	Yes	37 (9.1)
Tumour perforation^f^	No	404 (98.5)
	Yes	6 (1.5)
Surgical complication^i^	No	209 (56.6)
	Yes	160 (43.4)

^a^n = 336, ^b^n = 298, ^c^n = 405, ^d^n = 393, ^e^n = 404, ^f^n = 410, ^g^n = 409, ^h^n = 408, ^i^n = 369.

The relationship between clinicopathological characteristics and both composite ratios and cumulative scores is shown in Table 3 (n = 413). Both composite ratios and cumulative scores showed a correlation with neoadjuvant therapy (p < 0.001) and time of surgery (p < 0.001).

**Table 3:** The correlation between composite ratios and cumulative scores and clinicopathological characteristics of patients undergoing elective surgery for rectal cancer (n = 413).

	Age	Sex	BMI	ASA grade	T-stage	N-stage	Differentiation	Venous invasion	Margin involvement	Peritoneal involvement	Tumour perforation	Neoadjuvant therapy	Adjuvant therapy	Time of surgery	Surgical complication
NLR	0.349	0.003	0.050	0.053	0.269	0.234	0.952	0.357	0.007	0.338	0.106	< 0.001	0.466	0.871	0.235
NLS	0.265	0.539	0.024	0.018	0.044	0.785	0.096	0.442	0.019	0.400	0.006	< 0.001	0.646	0.738	0.974
PLR	0.662	0.972	0.004	0.797	0.216	0.366	0.838	0.027	0.184	0.507	0.203	< 0.001	0.724	0.697	0.174
PLS	0.081	0.702	0.010	0.044	0.058	0.559	0.110	0.406	0.042	0.330	0.147	< 0.001	0.319	0.708	0.631
LMR	0.600	0.005	0.005	0.019	0.126	0.063	0.688	0.068	0.066	0.227	0.154	< 0.001	0.319	< 0.001	0.976
LMS	0.768	0.008	0.008	0.471	0.080	0.864	0.007	0.618	0.122	0.137	0.045	< 0.001	0.320	< 0.001	0.914
NPS	0.683	0.023	0.023	< 0.001	0.024	0.885	0.225	0.119	0.002	0.022	0.230	0.944	0.030	0.046	0.664
CAR	0.787	0.873	0.873	0.002	0.005	0.919	0.727	0.262	0.060	0.001	0.772	0.671	0.192	< 0.001	0.672
mGPS	0.893	0.259	0.259	< 0.001	0.009	0.596	0.522	0.140	0.019	0.005	0.215	0.766	0.516	0.131	0.598

*p < 0.001 considered significant.

The relationship between composite ratios and cumulative scores and their component values were shown in Table 4 (n = 413). Both ratios and scores showed that a majority of patients were not systemically inflammed prior to surgery (NLR > 5 18%, NLS ≥ 1 48%, PLR > 150 58%, PLS ≥ 1 48%, NPS ≥ 1 11%, CAR > 0.22 28%, mGPS ≥ 1 24%).

**Table 4:** The relationship between composite ratios and cumulative scores and their component values in patients undergoing surgery for rectal cancer (n = 413).

	n (%)	Median (range)	
		Neutrophil	Lymphocyte
**NLR**			
≤ 3	209 (50.6)	4.0 (1.4–9.9)	2.0 (0.7–6.1)
3–5	130 (31.5)	5.1 (2.0–11.5)	1.3 (0.5–2.4)
> 5	74 (17.9)	6.0 (2.8–15.4)	0.8 (0.3–1.5)
**NLS**			
0	217 (52.5)	4.4 (2.1–7.5)	2.0 (1.5–6.1)
1	177 (42.9)	4.7 (1.4–11.5)	1.1 (0.3–3.8)
2	19 (4.6)	8.6 (7.6–15.4)	0.9 (0.3–1.4)

	n (%)	Median (range)	
		Platelet	Lymphocyte
**PLR**^**a**^			
≤ 150	151 (41.8)	234 (125–504)	2.1 (0.9–6.1)
> 150	210 (58.2)	277 (128–648)	1.2 (0.3–3.2)
**PLS**^**a**^			
0	187 (51.8)	263 (132–399)	2.0 (1.5–5.0)
1	164 (45.4)	255 (125–648)	1.1 (0.3–6.1)
2	10 (2.8)	426 (404–498)	1.1 (0.6–1.4)

	n (%)	Median (range)	
		Lymphocyte	Monocyte
**LMR**^**b**^			
≥ 2.4	133 (61.0)	1.8 (0.7–5.0)	0.5 (0.1–1.2)
< 2.4	85 (39.0)	1.0 (0.4–2.5)	0.6 (0.3–1.3)
**LMS**^**b**^			
0	105 (48.2)	1.9 (1.5–3.9)	0.6 (0.3–0.8)
1	106 (48.6)	1.0 (0.4–5.0)	5.0 (0.1–1.2)
2	7 (3.2)	0.9 (0.4–1.4)	1.0 (0.9–1.3)

	n (%)	Median (range)	
		Neutrophil	Platelet
**NPS**^**a**^			
0	320 (88.6)	4.4 (1.6–7.5)	254 (125–399)
1	33 (9.1)	7.8 (3.0–15.4)	362 (207–629)
2	8 (2.2)	8.5 (7.6–10.9)	465 (405–648)

	n (%)	Median (range)	
		CRP	Albumin
**CAR**			
≤ 0.22	298 (72.2)	4.2 (0.1–9.0)	39 (25–52)
> 0.22	115 (27.8)	19 (7.0–208)	36 (18–47)
**mGPS**			
0	314 (76.0)	5 (0.1–10.0)	39 (25–52)
1	58 (14.0)	20 (0.1–112)	39 (34–47)
2	41 (9.9)	24 (11–208)	32 (18–34)

^a^n = 361, ^b^n = 218.

The median values for the components of the composite ratios and cumulative scores are shown in Table 4. An NLR 3–5 was associated with a median neutrophil and lymphocyte count of 5.1 × 10^9^/l and 1.3 × 10^9^/l respectively, both within normal reference ranges. In contrast, an NLR > 5 was associated with a median neutrophil and lymphocyte count of 6.0 × 10^9^/l and 0.8 × 10^9^/l respectively, with lymphocytes being outside the normal reference range. A PLR > 150 was associated with a median platelet and lymphocyte count of 277 × 10^9^/l and 1.2 × 10^9^/l respectively, both within normal reference ranges. An LMR < 2.4 was associated with a median lymphocyte and monocyte count of 1.0 × 10^9^/l and 0.6 × 10^9^/l respectively, both within normal reference ranges. A CAR > 0.22 was associated with a median CRP concentration of 19 mg/l and a median albumin concentration of 36 g/l, CRP being outside the normal reference range.

No correlation between the systemic inflammatory response (mGPS and NPS) and neoadjuvant therapy was seen. In addition, Kaplan Meier analysis showed that there was no relationship between neoadjuvant therapy and survival in patients undergoing surgery between 1997 and 2015 (p = 0.872). Furthermore, additional Kaplan Meier analysis showed no relationship between neoadjuvant therapy and survival in patients undergoing surgery between 1997 and 2005 (p = 0.854) or between 2007 and 2015 (p = 0.466).

**Figure 1:**
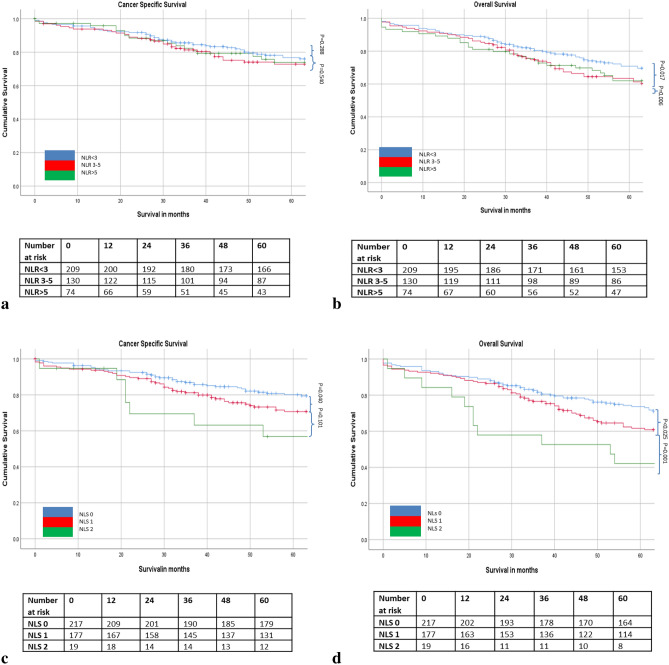
(**a**–**d**) The relationship between the NLR and NLS and both CSS and OS in patients undergoing surgery for rectal cancer. Number at risk depicts the number of patients alive or not censored entering each time period.

**Figure 2:**
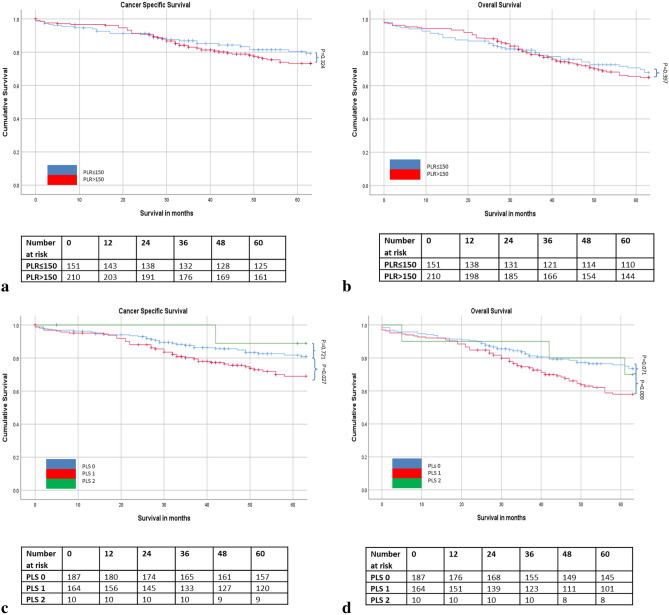
(**a**–**d**) The relationship between the PLR and PLS and both CSS and OS in patients undergoing surgery for rectal cancer. Number at risk depicts the number of patients alive or not censored entering each time period.

**Figure 3:**
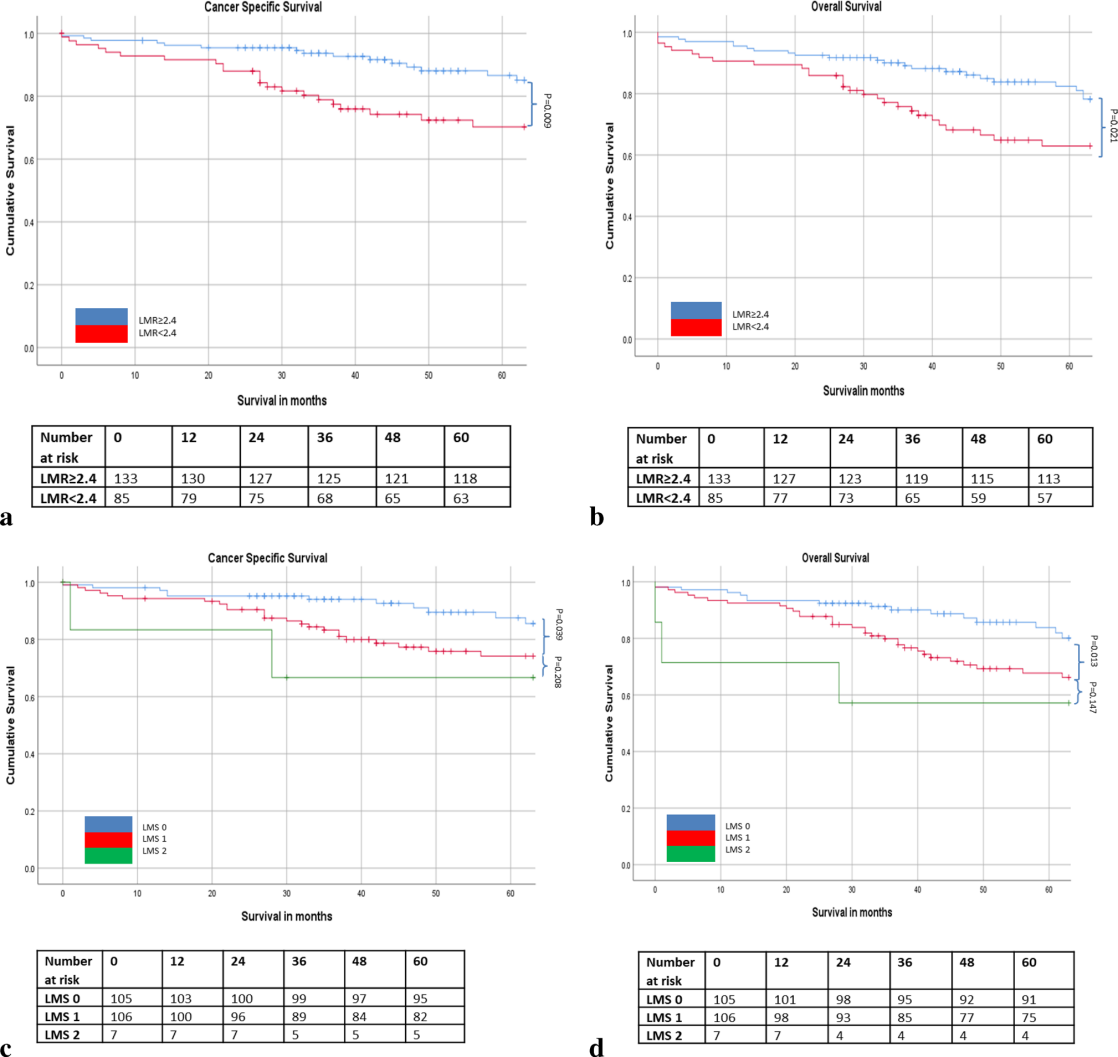
(**a**–**d**) The relationship between the LMR and LMS and both CSS and OS in patients undergoing surgery for rectal cancer. Number at risk depicts the number of patients alive or not censored entering each time period.

**Figure 4:**
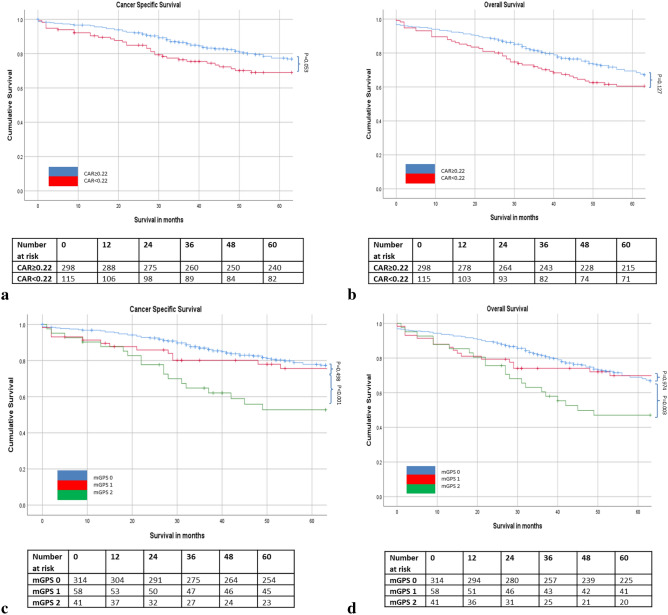
(**a**–**d**) The relationship between the CAR and mGPS and both CSS and OS in patients undergoing surgery for rectal cancer. Number at risk depicts the number of patients alive or not censored entering each time period.

The relationship between ratios, scores and 5 year cancer specific survival is shown in Table 5 and Figs. 1, 2, 3 and 4. On ROC analysis using cancer specific survival as an end-point the AUC for TNM stage was 0.622, Type of Surgery was 0.575, Time of Surgery was 0.608, NLR was 0.525, NLS was 0.565, PLR was 0.535, PLS was 0.566, LMR was 0.559, LMS was 0.612, NPS was 0.538, CAR was 0.554 and mGPS was 0.577. When adjusted for TNM stage, Type of Surgery, Time of Surgery and Margin Involvement mGPS 2 (p < 0.05) remained associated with cancer specific survival.

**Table 5:** The relationship between validated ratios, scores and survival in patients undergoing surgery for rectal cancer (n = 413).

	ROC-AUC	Univariate		Multivariate Adjusted for TNM stage, Type of Surgery and Time of Surgery, Margin Involvement		ROC-AUC	Univariate		Multivariate Adjusted for TNM stage, Type of Surgery and Time of Surgery, Margin Involvement	
		CSS HR (95% CI)	p-value	CSS HR (95% CI)	p-value		OS HR (95% CI)	p-value	OS HR (95% CI)	p-value
**TNM stage**										
I (n = 88)	0.622 (0.564–0.680)					0.596 (0.542–0.651)				
II (n = 161)		2.05 (1.06–3.98)	0.034	1.85 (0.89–3.85)	0.098		1.39 (0.89–2.15)	0.144	1.24 (0.75–2.05)	0.395
III (n = 164)		3.22 (1.70–6.13)	< 0.001	2.64 (1.29–5.40)	0.008		1.89 (1.23–2.91)	0.004	1.65 (1.01–2.69)	0.045
**Type of surgery**										
Open (n = 343) vs laparoscopic (n = 70)	0.575 (0.517–0.634)	2.51 (1.16–5.41)	0.019	2.05 (0.94–4.67)	0.070	0.614 (0.560–0.667)	2.48 (1.30–4.72)	0.006	1.84 (0.95–3.60)	0.073
**Time to surgery**										
1997–2004 (n = 129)	0.608 (0.547–0.669)					0.698 (0.647–0.749)				
2005–2015 (n = 284)		0.76 (0.52–1.10)	0.146	0.76 (0.49–1.18)	0.215		0.64 (0.47–0.87)	0.005	0.74 (0.53–1.05)	0.094
**Margin involvement**										
R0 (n = 364)	0.564 (0.499–0.628)					0.532 (0.475–0.588)				
R1 (n = 45)		2.83 (1.79–4.48)	< 0.001	2.59 (1.59–4.21)	< 0.001		1.88 (1.26–2.82)	0.002	1.76 (1.14–2.72)	0.010
**Surgical complication**										
No (n = 209)	0.556 (0.490–0.622)					0.531 (0.472–0.590)				
Yes (n = 160)		1.61 (1.09–2.38)	0.017	1.39 (0.94–2.07)	0.104		1.50 (1.10–2.04)	0.010	1.49 (1.07–2.07)	0.017
**NLR/ NLS**										
NLR < 3 (n = 209)	0.525 (0.463–0.587)					0.595 (0.540–0.649)				
NLR 3–5 (n = 130)		1.25 (0.83–1.89)	0.288	1.18 (0.76–1.84)	0.465		1.44 (1.04–1.99)	0.027	1.45 (1.02–2.05)	0.038
NLR > 5 (n = 74)		1.17 (0.71–1.92)	0.540	0.82 (0.47–1.44)	0.494		1.68 (1.17–2.40)	0.005	1.32 (0.87–1.98)	0.188
NLS 0 (n = 217)	0.565 (0.503–0.627)					0.585 (0.530–0.640)				
NLS 1 (n = 177)		1.49 (1.02–2.17)	0.040	1.40 (0.92–2.12)	0.115		1.39 (1.04–1.87)	0.025	1.33 (0.97–1.84)	0.077
NLS 2 (n = 19)		1.94 (0.88–4.28)	0.101	1.73 (0.76–3.95)	0.191		2.52 (1.46–4.37)	0.001	2.21 (1.22–4.03)	0.009
**PLR/ PLS**										
PLR ≤ 150 (n = 151)	0.535 (0.468–0.603)					0.532 (0.472–0.592)				
PLR > 150 (n = 210)		1.24 (0.81–1.89)	0.324	1.16 (0.73–1.83)	0.536		1.15 (0.83–1.58)	0.403	1.08 (0.76–1.53)	0.676
PLS 0 (n = 187)	0.566 (0.499–0.634)					0.594 (0.535–0.653)				
PLS 1 (n = 164)		1.60 (1.06–2.43)	0.027	1.36 (0.87–2.14)	0.183		1.56 (1.13–2.15)	0.007	1.36 (0.96–1.94)	0.083
PLS 2 (n = 10)		1.24 (0.38–4.01)	0.721	1.06 (0.32–3.57)	0.113		2.01 (0.96–4.18)	0.063	1.47 (0.62–3.50)	0.381
**LMR/ LMS**										
LMR ≥ 2.4 (n = 133)	0.559 (0.445–0.673)					0.589 (0.505–0.673)				
LMR < 2.4 (n = 85)		2.22 (1.22–4.04)	0.009	2.08 (1.13–3.845)	0.019		1.78 (1.09–2.91)	0.021	1.73 (1.05–2.83)	0.031
LMS 0 (n = 105)	0.612 (0.517–0.706)					0.592 (0.492–0.692)				
LMS 1 (n = 106)		1.97 (1.03–3.74)	0.039	1.90 (0.99–3.63)	0.054		1.97 (1.16–3.37)	0.013	2.01 (1.17–3.44)	0.011
LMS 2 (n = 7)		2.60 (0.59–11.47)	0.208	2.38 (0.54–10.59)	0.255		2.47 (0.73–8.35)	0.147	2.51 (0.74–8.51)	0.139
**NPS**										
NPS 0 (n = 320)	0.538 (0.468–0.607)					0.569 (0.509–0.630)				
NPS 1 (n = 33)		2.23 (1.28–3.88)	0.005	1.72 (0.92–3.22)	0.087		2.03 (1.30–3.16)	0.002	1.97 (1.22–3.16)	0.005
NPS 2 (n = 8)		0.58 (0.08–4.13)	0.582	0.70 (0.10–5.07)	0.727		2.55 (1.19–5.48)	0.016	2.40 (0.97–5.94)	0.058
**CAR/ mGPS**										
CAR ≤ 0.22 (n = 298)	0.554 (0.491–0.617)					0.549 (0.494–0.605)				
CAR > 0.22 (n = 115)		1.46 (0.99–2.14)	0.053	1.14 (0.75–1.75)	0.541		1.26 (0.94–1.70)	0.127	1.13 (0.81–1.56)	0.483
mGPS 0 (n = 314)	0.577 (0.513–0.641)					0.553 (0.497–0.608)				
mGPS 1 (n = 58)		1.20 (0.71–2.03)	0.498	1.11 (0.64–1.94)	0.701		1.03 (0.69–1.54)	0.879	1.06 (0.69–1.61)	0.796
mGPS 2 (n = 41)		2.59 (1.61–4.17)	< 0.001	2.09 (1.17–3.72)	0.012		1.79 (1.19–2.69)	0.005	1.56 (0.96–2.53)	0.074

On ROC analysis using overall survival as an end-point the following AUC for TNM stage was 0.596, Type of Surgery was 0.614, Time of Surgery was 0.698, NLR was 0.595, NLS was 0.585, PLR was 0.532, PLS was 0.594, LMR was 0.590, LMS was 0.589, NPS was 0.569, CAR was 0.549 and mGPS was 0.553. When adjusted for TNM stage, Type of Surgery, Time of Surgery and Margin Involvement NLR 3–5 (p < 0.05), NLS 2 (p < 0.05), LMR < 2.4 (p < 0.05), LMS 1 (p < 0.05) and NPS 1 (p < 0.05), and mGPS 2 (p < 0.01) remained associated with overall survival (Table 5 and Figs. 1, 2, 3 and 4).

The prognostic values of the NPS and mGPS were examined in the context of TNM staging in Table 6. Within TNM stage II disease the 5-year cancer specific survival rate was 74%. This varied between 77% and 59% according to the NPS and between 76% and 68% according to the mGPS. The 5-year overall survival rate was 56%. This varied between 62% and 23% according to the NPS and between 60% and 46% according to the mGPS.

**Table 6:** The relationship between mGPS, NLS and 5 year cancer specific survival (CSS) and overall survival (OS) rates in patients undergoing potentially curative resection of TNM stage II (n = 137) and III (n = 140) rectal cancer.

	mGPS 0		mGPS 1/2		mGPS 0–2		mGPS 0		mGPS 1/2		mGPS 0–2	
	n	5 year CSS % (SE)	n	5 year CSS % (SE)	n	5 year CSS (%)	n	5 year OS % (SE)	n	5 year OS % (SE)	n	5 year OS % (SE)
NPS 0	83 (89%)	77.1 (0.05)	32 (73%)	75.0 (0.08)	115	76.5 (0.04)	83 (89%)	63.9 (0.05)	32 (73%)	56.3 (0.09)	115	61.7 (0.05)
NPS 1/2	10 (11%)	70.0 (0.15)	12 (27%)	50.0 (0.15)	22	59.1 (0.11)	10 (11%)	30.0 (0.15)	12 (27%)	16.7 (0.11)	22	22.7 (0.09)
NPS 0–2	93	76.3 (0.04)	44	68.2 (0.07)	137	73.7 (0.04)	93	60.2 (0.05)	44	45.5 (0.08)	137	55.5 (0.04)
	Stage III (n = 140)						Stage III (n = 140)					
NPS 0	106 (93%)	70.8 (0.04)	18 (69%)	66.7 (0.11)	124	70.2 (0.04)	106 (93%)	57.5 (0.05)	18 (69%)	44.4 (0.12)	124	55.6 (0.05)
NPS 1/2	8 (7%)	75.0 (0.16)	8 (31%)	37.5 (0.18)	16	56.3 (0.13)	8 (7%)	37.5 (0.18)	8 (31%)	25.0 (0.16)	16	31.3 (0.12)
NPS 0–2	114	71.1 (0.04)	26	57.7 (0.10)	140	68.6 (0.04)	114	56.1 (0.05)	26	38.5 (0.10)	140	52.9 (0.04)

Values are expressed as % (standard error: SE).

Within TNM stage III disease the 5 year cancer specific survival rate was 69%. This varied between 70 and 56% according to the NPS and between 71 and 58% according to the mGPS. The 5 year overall survival rate was 53%. This varied between 56 and 31% according to the NPS and between 56 and 39% according to the mGPS (Table 6).

When those patients who received neoadjuvant treatment were considered only 34 patients died of their cancer on follow up. This precluded further meaningful statistical analysis.

## Discussion

This study for the first time directly compares the prognostic value of measuring the systemic inflammatory response with composite ratios and cumulative scores in patients undergoing surgery for rectal cancer. The results are very similar to those reported by Dolan and co-workers in patients with colon cancer which showed that both composite ratios and cumulative scores had prognostic value, independent of TNM stage^6^. Furthermore, when cumulative scores and composite ratios constructed from lymphoid/myeloid tissue or from acute phase proteins were compared directly they had similar prognostic value^6^. When these results are interpreted together they highlight the importance of active monitoring of the systemic inflammatory response in patients with rectal cancer.

**Figure 5:**
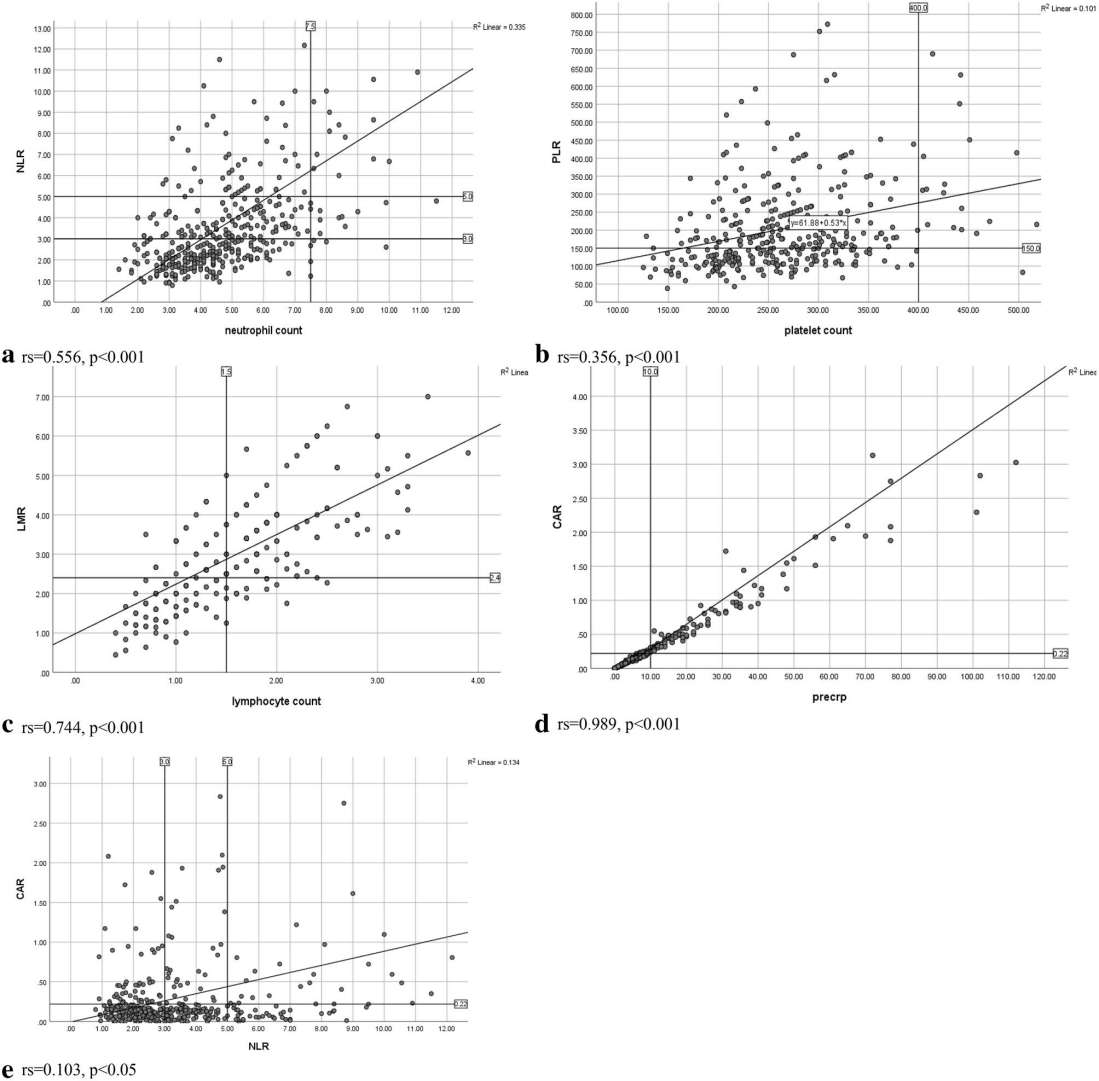
(**a**–**e**) Plot of preoperative neutrophil count and NLR, platelet count and PLR, lymphocyte count and LMR, CRP and CAR, NLR and CAR in all patients undergoing surgical resection for rectal cancer.

As has previously been found in colon cancer, ROC curve derived thresholds did not always differentiate normal and abnormal values of the individual component parts of the ratio^6^. Indeed, as can be seen in Fig. 5a when a line of best fit was applied, an NLR > 5 and an NLR > 3 were associated with a median neutrophil count of approximately 6.0 and 4.0 respectively, both within the normal reference range. Similar results were found for a PLR > 150 and an LMR < 2.4 which were associated with a platelet count of approximately 175 × 10^9^/l and a lymphocyte count of 1.2 × 10^9^/l, both within the normal reference range (Fig. 5b,c). As a result, it is apparent that a number of composite ratios contain individual readings within normal reference ranges. In addition, when compared with cumulative scores, composite ratios classify more patients as being systemically inflamed (Table 5).

As was the case in patients with colon cancer the results of this study provide further evidence for discontinuing the use of composite ratios to monitor the systemic inflammatory response in patients with rectal cancer^6^. Indeed, when composite ratios of lymphoid and myeloid cells (NLR) and acute phase proteins (CAR) are plotted against each other (Fig. 5e) they do not follow a mirror image pattern. In contrast, when cumulative scores of the differential white cell count (NPS) and acute phase proteins (mGPS) were compared directly they showed closer agreement for both their systemic inflammatory response status and prognostic value (Table 6). Therefore, the use of cumulative score, based on normal reference ranges, builds on a fuller understanding of activation of the innate systemic inflammatory response seen in patients with colon cancer^6^. In addition cumulative scores have a greater level of simplicity and consistency which adds to their clinical relevance in patients with rectal cancer.

Furthermore, a clear correlation between factors associated with poorer survival such sex, BMI, advanced T-stage and ASA grades were seen for both higher composite ratios and cumulative scores. This adds further weight to the prognostic strength of cumulative scores in particular in patients with rectal cancer. It is of interest that there was significant correlation between cumulative scores and composite ratios and patients who underwent neoadjuvant chemoradiotherapy. This would follow current European clinical practice to offer neoadjuvant chemoradiotherapy to patients with locally advanced cancers with an associated increased tumour load and inflammatory response. It would also suggest that routine monitoring of the systemic inflammatory response will continue to be of increasing importance as management of rectal cancers moves away from a blanket surgical approach to one where watchful waiting plays a much more significant part^3^.

The present study has a number of possible limitations. Although a relatively large prospective cohort, there were small numbers of observations in some sub-group analysis. In addition the time scale of the study crosses the change in standard chemotherapy regimes in 2005 to an oxaliplatin based treatment standard. However, it is interesting that when entered into cox-regression analysis time of surgery did not prove to be prognostic. Furthermore, data relating to other factors that may have affected markers of the systemic inflammatory response such drugs taken prior to sampling were not available. Prior work performed by Sung^11^ and Lee^12^ have shown the predictive values of the NLR & PLR in both pre and post-CRT. Therefore, it would have been interesting to carry out further subgroup analysis to assess the prognostic value of both ratios and scores in the pre neoadjuvant CRT setting in addition to the pre-operative timepoint. However, the available data did not allow for this. Given that cancer related inflammation correlates with poor outcomes^13,14^, the hope would be that further characterisation of composite ratios will enable a better prediction model to determine those patients who are more likely to have a palliative colorectal cancer as tumour size has been previously shown by De Felice and co-workers^15^.

In summary, this study for the first time directly compares the prognostic value of measuring the systemic inflammatory response with composite ratios and cumulative scores in patients undergoing surgery for rectal cancer. The results complement those of a recent study by Dolan and co-workers in colon cancer and show that both composite ratios and cumulative scores had prognostic value, independent of TNM stage, surgical approach, complications and margin involvement^6^. However, as was the case in patients with colon cancer, cumulative scores are simpler to construct and more consistent for clinical use^6^. Direct comparison of the performance of the two variables would be required to definitively characterise their prognostic accuracy in clinical use, however these results would suggest that mGPS could be used in the initial workup of patients undergoing neoadjuvant therapy for rectal cancer. They could also guide those patients who should be considered for surgical resection irrespective of their response to neoadjuvant therapy.
